# Viability of *Cabralea canjerana* extracts to control the South American fruit fly, *Anastrepha fraterculus*

**DOI:** 10.1093/jis/14.1.47

**Published:** 2014-01-01

**Authors:** Flaviane Eva Magrini, Alexandre Specht, Juliano Gaio, Cristiane Priscila Girelli, Ignacio Migues, Horacio Heinzen, Valdirene Camatti Sartori, Veronica Cesio

**Affiliations:** 1 Universidade de Caxias do Sul – Instituto de Biotecnologia, Laboratório de Controle de Pragas, Caxias do Sul, RS, Brasil 95070-560; 2 Embrapa Cerrados, BR 020 Km 18 Planaltina, DF, Brasil 73310-970; 3 Universidad de la Republica, Facultad de Química, Laboratorio de Quimica de Productos Naturales, Montevideo,Uruguai (11800)

**Keywords:** antifeedant activity, toxic activity, oviposition deterrence

## Abstract

Several representatives of Meliaceae contain biologically active compounds that are toxic to insects with few negative effects on the environment and humans. Our study evaluated the activity of ethyl acetate and ethanol extracts from the fruit and seeds of *Cabralea canjerana* (Vellozo) Mart (Sapindales: Meliaceae) on *Anastrepha fraterculus* (Wiedemann) (Diptera: Tephritidae). Limonoids and triterpenes were detected in fruit and seed extracts. Each extract was added to an artificial diet at three concentrations and tested after 24, 48, and 72 hr of extract application. Ethyl acetate extracts were the most active ones and showed the effect of both dose and time elapses after application on the insects. The highest toxic effect on *A. fraterculus* adults was from ethyl acetate extracts from fruit, followed by extracts from seeds. These extracts showed antifeedant activities. Extract solutions sprinkled on fruits of *Carica papaya* (L.) (Brassicales: Caricaceae) caused oviposition repellency and negatively affected the biological development of *A. fraterculus* . Ethyl acetate extracts highly hampered oviposition, but seed extracts showed lesser oviposition deterrence. The fruit and seed extracts diminished pupal viability. Particularly, the ethyl acetate fruit extract caused malformed adults. The sex ratio was also affected, resulting in female predominance for the fruit extract, while the seed extract showed a dose-dependent effect. Low doses caused male abundance, but at higher concentrations the effect was reversed. These encouraging results showed that the *C. canjerana* extracts have great potential as new tools to be used in integrated pest management programs to protect fruits against *A. fraterculus* .

## Introduction

The South American fruit fly, *Anastrepha fraterculus* (Wiedemann) (Diptera: Tephritidae), is a key pest of many fruits in Argentina, Uruguay, and the southern and southeastern regions of Brazil ( Malavasi et al. 2000 ). Larvae of this fly develop in fruits of more than 67 native and cultivated hosts ( Zucchi 2000 ) and may compromise up to 100% of fruit production ( Carvalho 2006 ). The control of fruit fly adults and larvae is normally carried out with toxic baits. Unfortunately, the chemical compounds ( Kovaleski et al. 2000 ) in these baits not only kill the pest insects, but also harm the applicators and the environment. Residues from these baits contaminate the soil and consequently the food that grows on it, cause biological unbalance, and determine the selection of resistant populations ( Whalon et al. 2008 ). Alternatively, in organic orchards, essential oils, plant extracts, and soaps may be used ( Steffens and Schmutterer 1982 ; Stark et al. 1990 ; Prokopy and Powers 1995 ; Van-Randen and Roitberg 1998a , b; Salles and Rech 1999 ; Singh 2003 ; Khan et al. 2007 ; Ali et al. 2011 ; Efrom et al. 2011 ).

Hundreds of limonoids have been isolated from Meliaceae ( Connolly 1983 ; Viegas Jr. 2003 ). These bioactive compounds act in different ways against different insect orders ( Mordue and Nisbet 2000 ). Among the main known Meliaceae are the neem, *Azadirachta indica* ( Schmutterer 1990 ; Bostid 1992 ); the chinaberry tree, *Melia azedarach* ( Carpinella et al. 2002 ); cedar, *Cedrela fissilis* ( Ambrozin et al. 2006 ); andiroba, *Carapa guianensis* ( Lavie et al. 1973 ; Qi et al. 2004 ); *Dysoxylum malabaricum* ( Govindachari et al. 1994 , 1995); *Sandoricum koetjape* ( Powell et al. 1991 ); representatives of *Trichilia* ( Roel et al. 2000 ; Matos et al. 2009 ; Baatile et al. 2011 ); and *Cabralea canjerana* (Vellozo) Mart. (Sapindales: Meliaceae) ( Rao et al. 1975 ; Schmeda-Hirschmann et al. 1992 ; Braga et al. 2006 ; Sarria et al. 2011 ).

*Cabralea canjerana* is a perennial Meliaceae tree that occurs from Costa Rica to northern Argentina ( Pennington and Styles 1975 ; Barreiros and Souza 1986). Several therapeutic properties of this plant have been exploited, mainly by indigenous populations ( Bueno et al. 2005 ). From *C. canjerana* stems and seeds, limonoids of the gedunin group, mexicanolids ( Rao et al. 1975 ), and dammarane triterpenes have been described ( Rao et al. 1975 ; Braga et al. 2006 ; Sarria et al. 2011 ). Schmeda-Hirschmann et al. (1992) described the insecticidal activity of hexane and ethanol extracts of leaves and fruits of *C. canjerana* against nymphs of *Rhodnius neglectus* , one of the vectors of the Chagas disease. Recently, Sarria et al. (2011) described the effects of triterpenes and ocotilone limonoids, ca-braleadiol, angolensate, and 3-methyl-βdeacetylfissinolide isolated from ethanolic extracts of fruits and seeds against the fall armyworm, *Spodoptera frugiperda* . Our study aimed to evaluate the activity of ethanolic and ethyl acetate extracts obtained from fruits and seeds of *C. canjerana* on the ingestion and oviposition by adults and immatures of *A. fraterculus* .

## Materials and Methods

### Vegetal material

Fruits of *C. canjerana* were collected in December 2009 from trees growing within the campus of the University of Caxias do Sul (UCS), Caxias do Sul, Brazil (29°9'46"S and 51°8'52"W). The material was identified and classified by the plant taxonomist Dr. Ronaldo Adelfo Wasun at the Museum of Natural Sciences of the UCS. The voucher specimen (in HUCS # 35647) was deposited in the herbari-um of this museum.

### Plant extract preparation and identification of compounds

The seeds were manually separated from the fruits ( Cañigueral et al. 1998 ). The pericarp was cut into small pieces (1 cm ^3^ ), weighed (100 g), and homogenized in hexane (1:3) at room temperature for 30 min with magnetic stirring. Then, the solution was filtered and the hexane extract was discarded. The remaining fruits were extracted sequentially with ethyl acetate and ethanol (1:10) after 48 hr stirring for each solvent. All extracts were filtered with Watman filter paper # 1. The filtrate was evaporated under reduced pressure. Seed extracts were prepared the same way as the fruit extracts. Ethyl acetate fruit and seed extracts were evaluated phytochemically by thin layer chromatography (Merck aluminium TLC silica gel 60 F254, Merck Millipore, www.merckmillipore.com ) using the following mobile phase: chloroform: methanol (97:3) with different dyeing reagents: Dragendorff, copper sulfate, vanillin sulfuric acid, sugars-specific dyeing reagent (diphenylamine:aniline:phosphoric acid/acetone), UV, fluorescence, and anisaldehyde ( Anonymous 1974 ; Wagner and Bladt 1996 ).

Typically, 15 µL of 1 mg/mL extract solutions were seeded in a thin layer chromatography and developed to 10 cm. As both extracts were seeded at the same concentration, rough visual comparison of the relative amounts of the compounds present in each extract could be performed.

### Insects and rearing methodology

The *A. fraterculus* used in the bioassays were obtained from insects reared in the Pest Control Laboratory, Institute of Biotechnology, University of Caxias do Sul. The specimens were maintained at 25 ± 3°C, 70 ± 10% RH, and a 12:12 L:D photoperiod. Adults were confined in cages (30 x 30 x 40) lined with cheesecloth, and larvae were reared on papaya, *Carica papaya* (L.) (Brassicales: Caricaceae), substrate. Other details of the methodology followed Salles (1992) and Machota Jr. et al. (2010) .

### Antifeedant activity of extracts on adults

The tests were free choice tests. Females and males of *A. fraterculus* that were 15–20 days old were deprived of food for 12 hr. Three pairs were placed in each cylindrical plastic cage (9 cm diameter x 10 cm height). Each cage was considered as a repetition, and each treatment consisted of seven repetitions. A solution of commercial hydrolyzed protein currently employed as an artificial feed attractant for fruit flies (Bio Anastrepha 3%, Bio Controle, www.biocontrole.com.br ) ( Scoz et al. 2006 ) was used as nourishing solution both in the control and the treatment. In each treatment, the extracts were included at 5 mg/mL as well as Ponceau red dye at 1gL ^-1^ concentration level (Sigma-Aldrich, www.sigmaaldrich.com ) ( Cruz et al. 1997 ; Scoz et al. 2004 ) to test flies’ positive feedings. The test solutions were offered through a roll of cotton inserted into a 4 mL container ( Scoz et al. 2004 ). The antifeedant activity was assessed 24 hr after the beginning of the experiment, and the percentage of antifeedant effect was calculated using the Obeng-Ofori (1995) formula:

AF = percentage of antifeedant effect

Nc = number of insects on the control after the exposure.

Nt = number of insects on the treatment after the exposure

### Toxic effect of the extracts to adults

This assay was performed as described in the previous section, but the choice possibility was eliminated. The extracts were used at the concentrations of 1, 2.5, and 5 mg/mL in the different treatments, with the addition of 1 g/L ^-1^ Ponceau red dye to the hydrolyzed protein (3%) solution. Only hydrolyzed protein with dye was used to rear the control group in separate cages. The number of dead insects was assessed 24, 48, and 72 hr after the beginning of the experiment. The determination of the lethal concentration (LC50) and lethal time (TL50) was performed using a Probit analysis ( Finney 1971 ). When insect mortality in the control group was over 10%, the mortality values were corrected using Abbott’s formula (1925) .

### Effect of extracts on oviposition

Ten *A. fraterculus* couples, 15 to 20 days old, kept in wooden cages covered with voile fabric (30 x 30 x 30 cm) were assayed in each treatment as well as in control trials. The reported result for each experiment is the average of five repetitions.

Typically, in a no choice assay, one papaya fruit ( *C. papaya* ) was placed in each cage. The fruits were sprayed with the extract diluted with water and tween at 1, 2.5, and 5 mg/mL concentration level. Only water and tween were sprayed on the control fruits. The fruits were divided in four, and each set of fruits was offered to the flies at different times after spraying (1, 24, 48, and 72 hr). The fruits were exposed to insects for 24 hr, then transferred and stored individually in plastic pots (1000 mL) containing expanded and crushed vermiculite and covered with plastic sheets wrapped in voile fabric. After 15 days, the fruit leftovers were removed, the vermiculite was sifted, and the number of pupae per fruit was investigated.

### Effect of the extracts on fruit fly development

Besides the number of insects per fruit, certain morphological parameters of the pupae and adults were also evaluated. The shape of the pupae was characterized as normal or larviform ( Hallman and Zhang 1997 ). Then, the specimens, individualized by fruit, were kept in pots containing moistened vermiculite until the emergence of the adults. In the first days after emergence, the adults were euthanized in a freezer (-17°C) to later identify their sex ( Zucchi et al. 2000 ) and to calculate the sex ratio ( Silveira Neto et al. 1976 ). The percentage of morphological deformities was also assessed in them.

### Statistical Analysis

The experimental data were analyzed using oneway ANOVA, whose means were compared by Duncan’s test ( *P* ≤0.05) using the transformed square-root average (x + 0.5). The means and standard deviations presented in the tables represent the original values.

## Results

**Figure 1.**
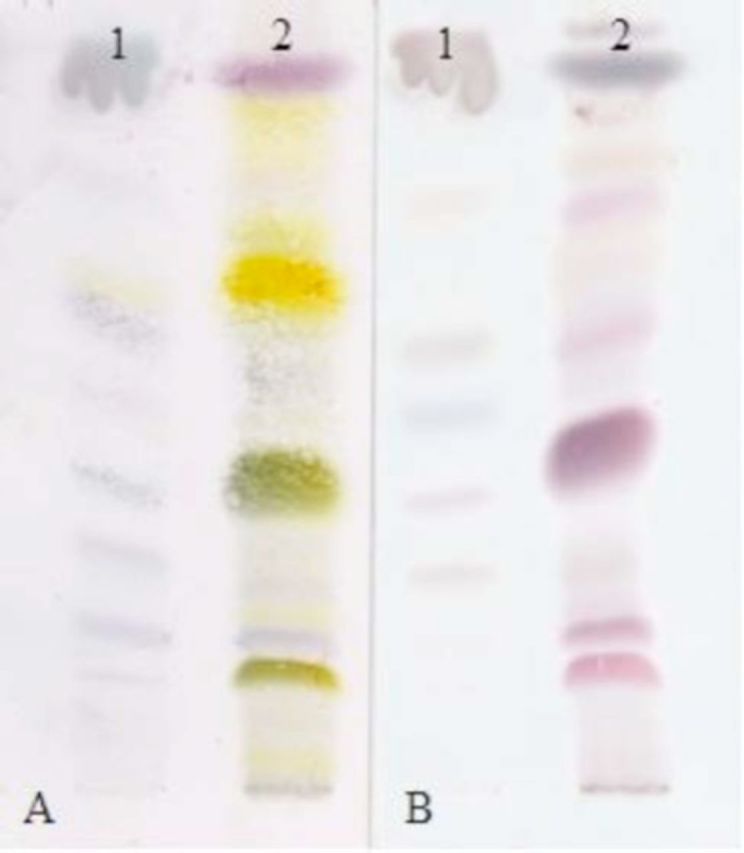
Thin layer chromatography of seed (1) and fruit (2) extracts of *Cabralea canjerana* obtained with ethyl acetate developed with vanillin sulfuric acid (A) and copper sulfate (B). High quality figures are available online.

The phytochemical study was performed following standard procedures ( Wagner and Bladt 1996 ) using thin layer chromatography as a tool to characterize the “digital fingerprint” of the extracts under study. Different dyeing reagents (Draggendorf, copper sulfate/phosphoric acid, vanillin/sulfuric acid, UV at 254 and 365 nm) were employed to obtain the chromatographic profiles for specific groups of secondary metabolites contained in the ethyl acetate fruit and seed extracts. The reported phytochemicals isolated from *C. canjerana* were dammarane type triterpenoids and phenolics, whose presence was confirmed in both extracts. After color development with copper sulfate and vanillin sulfuric acid reagents, their profiles were different ( Figure 1 ). The major compounds presented at Rf = 0.65 and Rf = 0.4 revealed yellow and green with vanillin sulfuric acid respectively, indicating the possible presence of lignans and oxidized terpenes in fruits. Lignans were not identified in the seed extract.

**Figure 2.**
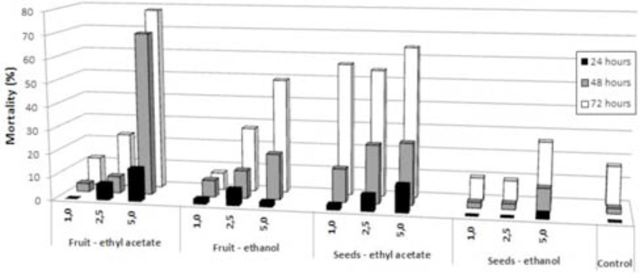
Cumulative mortality (%) of *Anastrepha fraterculus* adults offered an artificial diet (hydrolyzed protein Bio Anastrepha 3%) containing extracts of fruits and seeds obtained with ethyl acetate and ethanol in three concentrations (mg/mL), after 24, 48 and 72 hr exposure. High quality figures are available online.

All extracts of *C. canjerana* affected the survival of fruit fly adults ( Figure 2 ). The effect was more important when flies were exposed to fruits that were treated 48 to 72 hr before the beginning of the experiment. Even in the lack of a choice, flies did not feed the diet supplemented with extracts for at least 24 hr after the first exposure to the trial sample. However, we found that the flies in the control group fed immediately after being released into the cages. Both time and dose-dependent effects were observed in the mortality of *A. fraterculus* adults ( Figure 2 ). The most significant effects on mortality were associated with the ethyl acetate extracts of both fruits and seeds. They caused the highest mortality among fruit flies without statistical significance between them. Nevertheless, the smallest value for the median lethal concentration (LC50) was observed for the ethyl acetate seeds extract, but the lowest lethal time average (TL50) was observed for ethyl acetate fruit extract ( Table 1 ). These differences in pharmacokinetics as well as in the pharmacodynamics of the toxic action could be due to differences in the chemical profile of the extracts. On the other hand, the mortality induced by the ethanol extracts of fruits (1 mg/mL) and seeds (1 and 2.5 mg/mL) was less than 10% ( Figure 2 ).

**Table 1.**
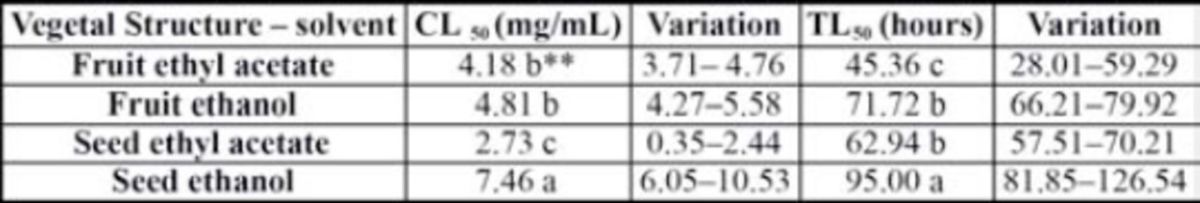
Mean lethal concentration LC50 and mean lethal time (TL50) of fruit and seed extracts of *Cabralea canjerana* , made with ethyl acetate and ethanol, incorporated to the artificial diet* of *Anastrepha fraterculus* adults.

*Hydrolyzed protein (Bio Anastrepha 3%). ** Means followed by distinct letters in each column are not significantly different according to the Duncan’s test *P* ≤ 0.05.

**Figure 3.**
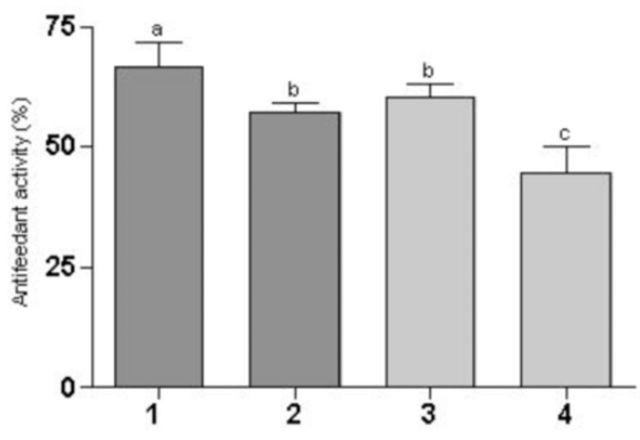
Mean percent antifeedant activity on *Anastrepha fraterculus* adults offered an artificial diet containing extracts in the concentration of 5 mg/mL and choice of (1) fruit ethyl acetate; (2) fruit ethanol; (3) seed ethyl acetate; (4) seed ethanol. High quality figures are available online

Three extracts showed good antifeedant activity towards *A. fraterculus* adults ( Figure 3 ). The ethyl acetate fruit and seed extracts as well as the ethanolic fruit extract showed 66– 57% antifeedant activity, whereas the ethanolic seed extract extracts had a 44% antifeedant activity. This antifeedant effect could be related to the toxicity observed for the ethyl acetate extracts. As the insects died, the antifeedant effect calculation shifts to higher values.

All extracts, at the evaluated concentrations, inhibited oviposition, especially in the early hours. There was also a dose-dependent effect observed. At higher concentrations, significant inhibition of oviposition was observed, even 72 hr after application of the extract, except for the ethanol seeds extract. However, at the highest concentration level, only the ethanol seeds extract completely repelled flies’ oviposition in the first 24 hr ( Table 2 ).

**Table 2.**
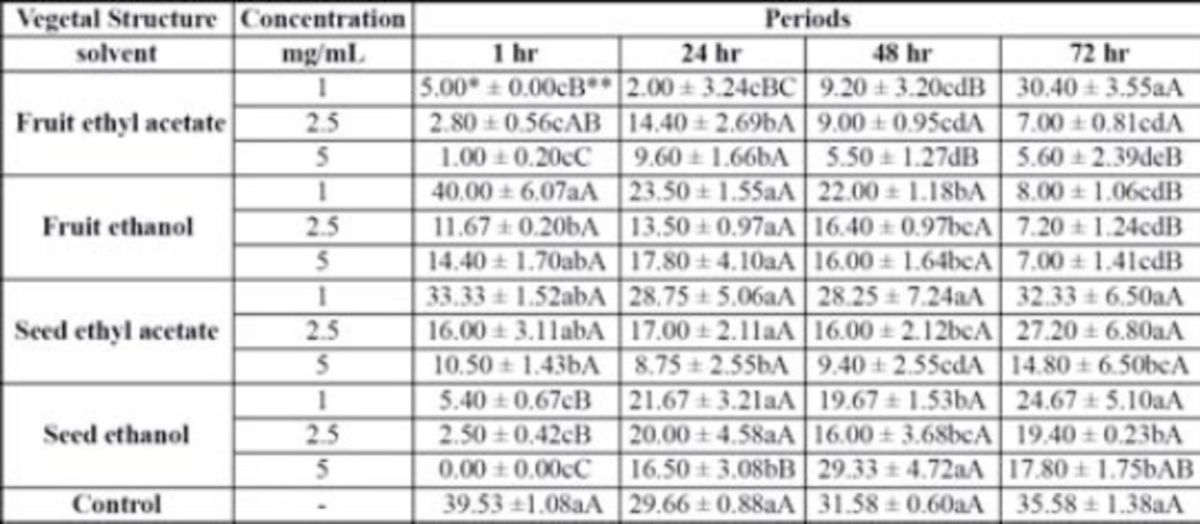
Mean number and standard error (SE) of *Anastrepha fraterculus* pupae per fruit ( *Carica papaya* ) pulverized with three concentrations of extracts of fruits and seeds of *Cabralea canjerana* , obtained with ethyl acetate and ethanol.

*Mean number of five replicates per treatment with ten adult couples of *Anastrepha fraterculus* aged 10 to 15 days. ** Means followed by the same letters are not significantly different (Duncan’s test *P* ≤ 0.05; lowercase letters for differences between extracts and concentrations; capital letters for differences between time intervals).

Besides the effects described and quantified above, a significant increase in the percentage of larviform pupae from papaya fruits sprayed with fruit extracts of *C. canjerana* was observed, especially at higher concentrations and during the first 24 hr ( Table 3 ).

**Table 3.**
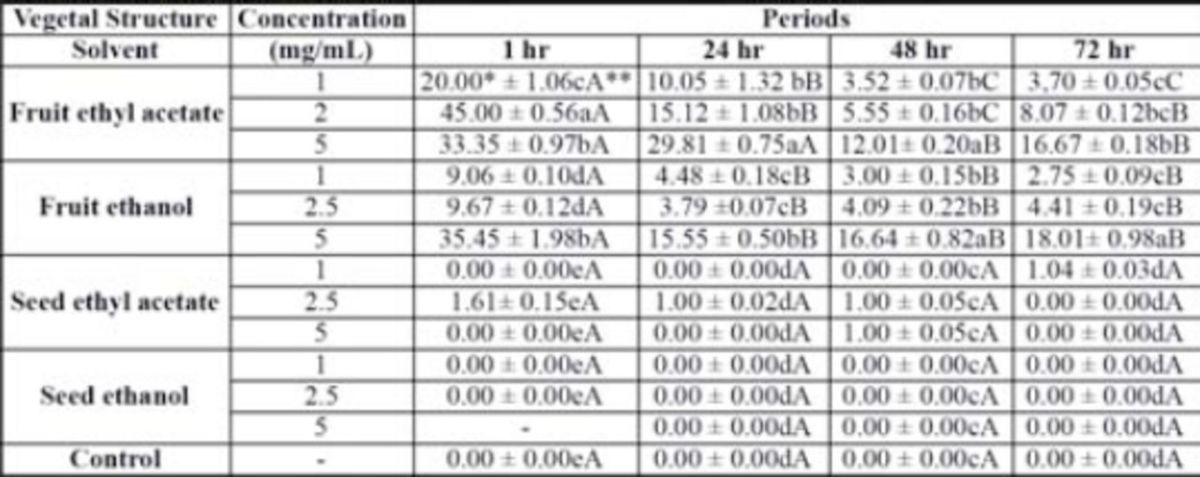
Percentage and standard error (SE) of *Anastrepha fraterculus* larviform pupae per fruit ( *C. papaya)* pulverized with different concentrations of *Cabralea canjerana* ethyl acetate and ethanol fruit and seed extracts.

*Mean number of five replicates per treatment with ten adult couples of *Anastrepha fraterculus* aged 10 to 15 days. **Means followed by the same letters are not significantly different (Duncan’s test *P* ≤ 0.05; lowercase letters for differences between extracts and concentrations; capital letters for differences between time intervals).

The pupal viability ( Table 4 ) of the insects from the treatments differed from that observed in the control, especially for the papaya fruit sprayed with *C. canjerana* fruits and seeds ethyl acetate extracts. When the highest concentration of this extract was used, it caused approximately 50% fruit fly mortality, and the effect lasted 72 hr. This residual effect was independent of the applied doses.

**Table 4.**
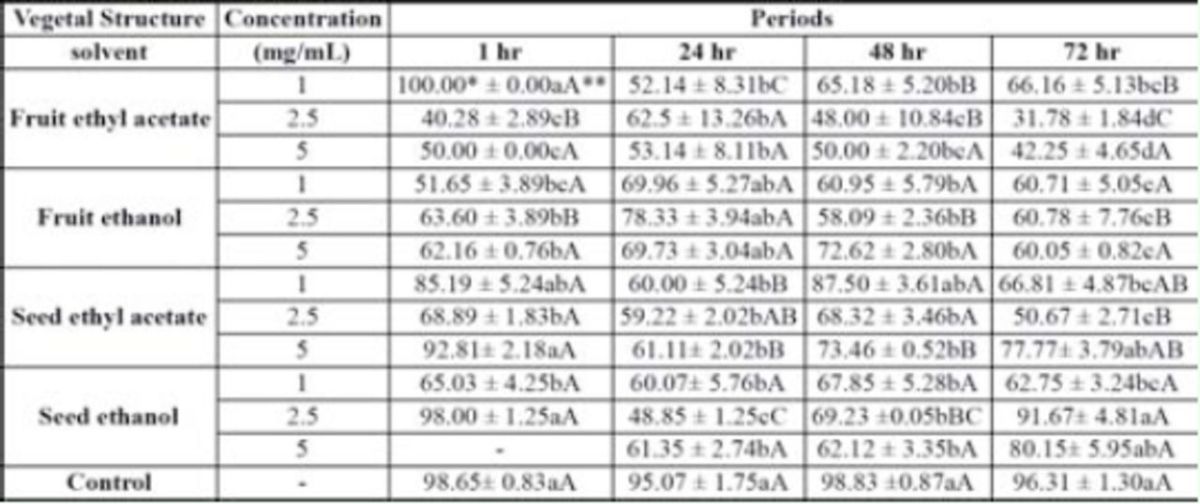
Mean percentage and standard error (SE) of pupal viability of *Anastrepha fraterculus* from fruits ( *Carica papaya)* pulverized with three concentrations of extracts of fruits and seed of *Cabralea canjerana* , obtained with ethyl acetate and ethanol

*Mean number of five replicates per treatment with ten adult couples of *Anastrepha fraterculus* aged 10 to 15 days. ** Means followed by the same letters are not significantly different (Duncan’s test *P* ≤ 0.05; lowercase letters for differences between extracts and concentrations; capital letters for differences between time intervals).

**Figure 4.**
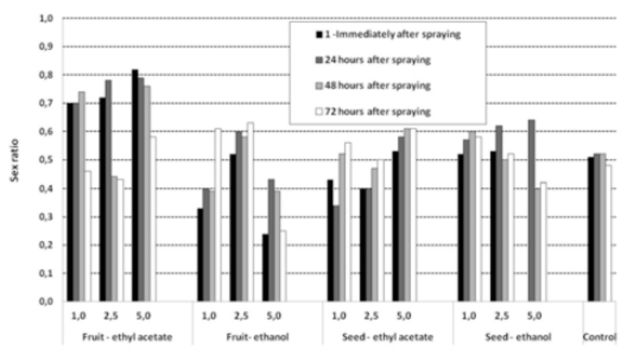
Sex ratio of flies that emerged from fruits of *Carica papaya* pulverized with different concentrations of *Cabralea canjerana* fruits and seed extracts, obtained with ethyl acetate and ethanol, in four distinct periodsHigh quality figures are available online.

The different extracts influenced the proportion of individuals of each sex in different ways. Overall, the sex ratio ( Figure 4 ) was skewed. Females prevailed in the treatments using higher concentrations of the extracts obtained with ethyl acetate. Males, by contrast, were more abundant when the ethanolic extract of fruits was used. These effects were kept throughout the 72-hr period.

**Figure 5.**
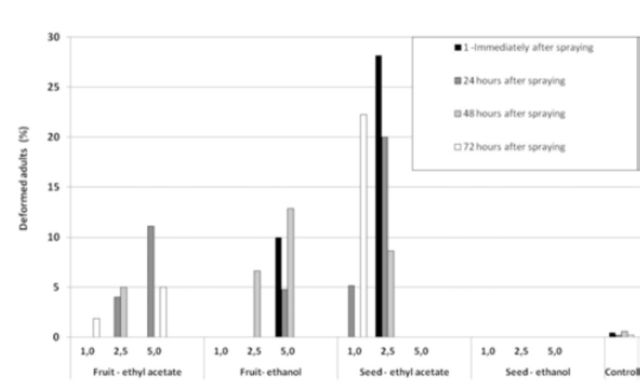
Mean number of flies with deformities that emerged from *Carica papaya* fruits pulverized with different concentrations of fruit and seed extracts of *Cabralea canjerana* , obtained with ethyl acetate and ethanol, in four distinct periods. High quality figures are available online.

The ethyl acetate seed extract caused the highest abnormalities ratio in emerging adults for all the extracts tested. Interestingly, ethanolic seed extracts caused no abnormalities at all. On the other hand, both fruit extracts caused adult abnormalities when compared to the control. Nevertheless, no direct relationship was observed between this occurrence and either concentration or time after spraying ( Figure 5 ). Abnormalities included incomplete emergence, lack of wings, stunted wings, no characteristic wing color, constricted body, oval body, and lack of antennae, mouthparts, and atrophied abdominal bulge.

## Discussion

The phytochemical evaluation revealed qualitative differences between the chromatographic profiles of the fruit and seed extracts, which may explain the different biological results obtained for the same bioassay. The colors observed with the vanillin/sulfuric acid reagent suggests the presence of both triterpenes and lignan type compounds, particularly the yellow spot detected with the latter that was not seen using copper sulfate-phosphoric acid, whereas the reddish-purple color that developed in these cases ( Figure 1 ) indicated the presence of limonoids and triterpenoids, which are the most common type of bioactive compounds reported for different representatives of Meliaceae ( Regnault-Roger et al. 2004 ; Hu et al. 2011 ), including Cabralea species, where the presence of dam-marane-type triterpenes in branches and stems was highlighted ( Braga et al. 2006 ).

*Cabralea canjerana* ethyl acetate fruit and seed extracts caused higher mortality rate to *A. fraterculus* than commercial neem oil (Botton et al. 2003; Efrom et al. 2011 ), but the sublethal effects were similar to those described previously ( Van-Randen and Roitberg 1998a ). Given that the mortality rate was directly correlated with the concentration of the extracts and the exposure period ( Figure 2 , Table 1 ), and therefore with volume ingested, we suggest that mortality of *A. fraterculus* was due to a cumulative process. It should be noted that, even in the absence of choice, flies within an experiment initially stopped eating, and just after 24 hr they restarted to feed on the fruit.

The antifeedant activity of the various extracts on *A. fraterculus* ( Figure 3 ) adults indicates that, like other Meliaceae, including neem ( Jacobson 1989 ; Schmetterer 1995; Senthil Nathan et al. 2005 , 2006; Coria et al. 2008 ; Alouani et al. 2009 ; Masood et al. 2009 ), *C. canjerana* also has various compounds that act as insect antifeedant agents. After ingestion of the test diet containing the extracts, the flies remained at the bottom of the cage in a lethargic state, not responding to external stimuli. This type of formulation, combining a feed attractant with the ethyl acetate seed extract, is an interesting alternative to control *Anastrepha* flies.

Many of the biological properties (larviform pupae, pupal viability, oviposition, number of eggs, deformed adults, sex distribution) described in our study are related to the well-known ecdisone-like properties of meliaceae triterpenoids and limonoids. These compounds are found mainly in the ethyl acetate extracts from *C. canjerana* due to their physicochemical properties. Ethanol extracts were less active, as they were obtained after the ethyl acetate extraction of the vegetal material, and little if any triterpenoid aglycones were left to be extracted.

The oviposition deterrence ( Table 2 ) induced by the extracts of fruits and seeds of *C. canjerana* in the no choice treatment was similar to that obtained in other studies with other Meliaceae species, where a reduction in the number of tephritid pupae was obtained with neem extracts ( Chen et al. 1996 ; Masood et al.2009 ; Ali et al. 2011 ).

The extracts inhibited the number of hatching eggs and/or turned eggs and larvae unviable after oviposition for at least 72 hr, effects that were also described by Van-Randen and Roitberg (1998b) . The high percentage of larviform pupae in the treatments ( Table 3 ), especially in insects that fed on the ethyl acetate fruit extract treatment, indicates that the triterpenoids and limonoids interfered with the hardening of the cuticle and the stabilization of protein structures ( Cruz 2000 ). These results are similar to the ones obtained with azadirachtin (1 mg/L), where its ingestion by newly-hatched larvae of *C. capitata* completelyprevented adult emergence ( Vinuela et al. 2000 ).

The lowest pupal viability ( Table 4 ) was found in the treatment with the ethyl acetate fruit extract. Pupal viability decrease has been reported in several studies that found a reduction in the survival of tephritids that had fed on meliceae extracts ( Steffens and Schmutterer 1982 ; Van-Randem and Roitberg 1998b; Vinuela et al. 2000 ; Mahfusa et al. 2007).

The variations in sex ratio ( Figure 4 ) associated with the different extracts indicate that these compounds acted differentially on individuals of each sex. As demonstrated before, several Meliaceae, especially neem, act on the hormone systems of insects ( Rembold 1995 ; Mordue (Luntz) and Nisbet 2000 ) and therefore have different effects on males and fe-females. This is the first study that addresses the differential effects of extracts on the immature stages and the sex ratio of fruit flies. These results indicate that more studies should be conducted detailing the effects of different substances and extracts on the development of males and females, especially on their reproductive systems.

Besides altering the viability and the number of larviform pupae, the extracts of fruits and seeds used in this study were responsible for an increase in the percentage of deformed adults ( Figure 5 ), especially the ethyl acetate seed extract. The abnormalities found in adults were consistent with those described in other studies involving Meliaceae and tephritids ( Steffens and Schmutterer 1982 ; Singh 2003 ; Silva et al. 2011 ).

The results of this study highlight toxic and deterrent properties (antifeedant and anti-oviposition) of extracts from *C. canjerana* to which *A. fraterculus* is vulnerable. These effects were observed for at least 72 hr. The different modes of action of the extracts of fruits and seeds of *C. canjerana* on *A. fraterculus* , particularly the antifeedant activity and oviposition deterrence for at least 72 hr, indicate their possible use in the integrated pest management of this fruit fly.
